# Evaluation of Cellular Fibronectin Plasma Levels as a Useful Staging Tool in Different Stages of Transitional Cell Carcinoma of the Bladder and Renal Cell Carcinoma

**Published:** 2007-02-07

**Authors:** A. Hegele, R. Hofmann, B. Kosche, J. Kropf

**Affiliations:** 1 Dept. of Urology/Pediatric Urology; 2 Dept. of Clinical Chemistry and Molecular Diagnostics; 3 Biomedical Research Center, Philipps University, Medical School, Marburg–Germany

**Keywords:** Extracellular matrix, TRFIA, Invasion, Biological markers, tumor progression

## Abstract

Reliable markers for both renal cell carcinoma (RCC) and transitional cell carcinoma of the bladder (TCC) are lacking.

During tumor progression and invasion components of extracellular matrix (ECM) are degraded and parts of these different components are detectable in plasma. Cellular fibronectin (cFN) represents a well characterized ECM protein. In contrast to fibronectin in plasma produced by hepatocytes (FN) cFN has a total extra domain sequence and occurs in much smaller amounts in the circulation. The aim of our study was to evaluate cFN as a marker and to determine its possible role in clinical staging of TCC and RCC.

Blood samples were collected from 30 patients before they underwent transurethral resection of the bladder because of newly diagnosed TCC. Additionally samples were collected from 69 patients with RCC before therapy. Sixty patients with non-malignant urological disorders were recruited as control group. Determination of cFN in plasma was performed by using a highly sensitive time-resolved fluorescence immunoassay (TRFIA).

The control group had median cFN plasma levels of 437 ng/ml. Patients suffering from TCC or RCC showed significantly higher cFN levels. In patients with muscle invasive TCC significant higher cFN levels (p < 0.05) could be demonstrated compared to non-muscle invasive TCC. Similar results were found in RCC with significant elevated cFN levels in metastatic RCC (p < 0.005) compared to localized stage of disease. No differences were found concerning tumor grading in both malignancies.

In the face of significant elevated cFN levels in TCC and RCC our data underline the important role of cFN. For future investigations the elevated cFN levels in locally progressed and metastastic disease, indicating a clinically useful tool for preoperative staging and postoperative monitoring, are of high interest.

## Introduction

Beside prostate cancer (PCA) transitional cell carcinoma of the bladder (TCC) and renal cell carcinoma (RCC) are the most frequent urological malignancies with rising incidence (Motzer and Russo, 2000; Huland H, 2006). For early detection, adequate stage adjusted therapy and risk adapted follow-up tumor markers would be a helpful clinical tool. A reliable tumor marker is only available for PCA since the introduction of prostate specific antigen. Various blood, urine and molecular markers have been described in TCC and RCC. But only for screening and monitoring patients with non-muscle invasive TCC FDA-approved urinary markers, like BTA- and NMP22 tests, are available (Giannopoulos et al. 2001; Boman H, et al. 2002). However, most of these potential markers have not reached clinical relevance due to low sensitivities, specificities or high costs (Zhao et al. 2005; Malats et al. 2005; Andreadis et al. 2005; Hegele et al. 2002; von Knobloch et al. 2002; Kashyap et al. 2005).

In TCC 75% of patients are initially presented with a superficial, 20% with muscle invasive and 5% with metastatic stage of disease (Heney, 1992). After primary treatment TCC recurs in 70% within a short time period and unfortunately about 20% will progress from superficial to advanced/metastatic stage of disease (Amling, 2001). In RCC we found a similar clinical dilemma. Twenty to thirty percent of primary localized disease will relapse despite adequate therapy and 30% are presented initially in a metastasized stage of disease with poor prognosis (Giberti et al. 1997; Whelan, 2003). In the face of these data it is of significant clinical interest to identify reliable markers for early detection and monitoring TCC and RCC after initial therapy.

Formation of metastases and local progression of tumors presuppose degradation of extracellular matrix (ECM) components. These essential steps are possible by hydrolytic enzymes of the tumor itself and induction by the tumor in the stromal cells (Danen, 2005; von Bredow et al. 1995). Products of this various ECM components degradation are released in the circulation and determination of these components can be helpful for early detection of several malignancies (Risteli and Risteli, 1995). Fibronectin (FN), a 440-KD glycoprotein, represents a well characterized ECM protein playing an important role in the inhibition of cellular attachment and tumor spread. The mechanism of FN action is mediated by specific receptors and growth factors (Danen, 2005; Hynes and Yamada, 1982). The main detectable fraction of FN in blood is synthesized by hepatocytes. In contrast cellular fibronectin (cFN) is found in much smaller amounts in the circulation. Cellular FN contains a specific extradomain sequence originating by differential splicing of the FN precursor mRNA. However, up to now the function of cFN is not clearly known (Warawdekar et al. 2006; Akiyama et al. 1995). Few studies described the potential role of cFN in different malignancies (Ylatupa et al. 1995; Haglund et al. 1997; Torbenson et al. 2002; Warawdekar et al. 2006). For example in hepatocellular carcinoma an over-expression of fibronectin protein was found (Torbenson et al. 2002). Elevated plasma levels were detected in patients suffering gastrointestinal and head/neck cancer (Warawdekar et al. 2006). In non-malignant diseases particularly in thrombosis, hemostasis, vascular disease and platelet function the definitive role of fibronectin is still unclear (Cho and Mosher, 2006; Mosher, 2006; Orem et al. 2002).

Recent studies in TCC and RCC indicating a potential role of cFN in these malignancies (Kirkali et al. 2001; Sanchez-Carbayo et al. 2000;Brenner et al. 2000). So further evaluation of cFN plasma levels, based on our first findings (Hegele et al. 2004; Hegele et al. 2002), is a consequent step to prove the clinical relevance in different stages of these malignancies and close this diagnostic gap.

## Material and Methods

To evaluate the value of cFN in different stages of TCC and RCC blood samples were collected in the morning before any therapy. Physical examination, chest X-ray, abdominal sonography and CT/MRT scan, where appropriate, were used for clinical staging. Patients showing other malignancies in medical history were excluded.

Blood samples were collected consecutively from 30 patients (13 women, 17 men, mean age 64 years) with newly diagnosed TCC without evidence of metastases before they underwent transurethral tumor resection over a time period of 4 months (March 2005–June 2005).

Additionally blood samples were collected from 45 consecutive patients (19 women, 26 men, mean age 69 years) with localized RCC before operative therapy over a time period of 10 months (September 2004–June 2005). Twenty-four patients (13 women, 11 men, mean age 63 years) with metastatic RCC before planned immunochemotherapy were also recruited in this time period. Out of the metastatic group 11 patients were initially presented with metastases and 13 developed metastatic disease during follow up after primary curative chirurgical intervention.

Sixty patients (30 women, 30 men, mean age 61 years) with benign urological disorders (for example stone disease, urinary tract infection, benign prostatic hyperplasia, urinary incontinence) and without malignancies in their medical history were consecutively recruited as control group.

Blood samples were collected in tubes coated with the potassium salt of ethylene diamine tetra-acetic acid (EDTA, Sarstedt, Germany) and processed for plasma within 3 hours. After centrifugation (2000 rpm, 10min, 4^o^C) and removal of supernatant plasma, the samples were stored at −70^o^C.

cFN was determined using a highly sensitive time-resolved fluorescence immunoassay (TRFIA). This sandwich-type assay, which is specific for the cellular form of FN, is based on a cFN-specific murine monoclonal antibody (provided by BIOHIT PLC, Finnland) immobilized on standard enzyme-linked immunosorbent assay microwells. Standards and samples were assessed in duplicate. A polyclonal FN-specific antiserum (rabbit) obtained from Sigma-Aldrich (Germany), was used as the secondary antibody. For the final detection step a modified biotin-streptavidin method with measurement of an europium-positive chelator was used (Kropf and Gressner, 1995; Kropf et al. 1991).

The assay has a linear measuring range from 12–3000 ng/ml and a detection limit of 4.0 ng/ml. Measurement of samples was always performed by the same person (B.K.).

The pathohistologic examination of the main tumor specimens was performed by the Institute of Pathology, Philipps-University Marburg, according to the classification of the International Union against Cancer 2002 (Wittekind et al. 2003). At least two experienced, board certified pathologist reviewed the specimens.

Statistical analysis was performed using the non parametric Mann-Whitney U test to compare the results of the malignancy groups with controls. The Kruskal-Wallis non-parametric ANOVA test was used to calculate the differences between the different groups and subgroups concerning TNM classification and nuclear grading (SPSS® software). A p-value <0.05 was accepted as statistically significant.

## Results

The intra-assay variance was evaluated by 20-fold determination of one sample. The coefficient of variation was 4.9%. The inter-assay variance was determined on 7 different days. The coefficient of variation was 6.9%.

The histological type of RCC was clear cell carcinoma in all patients. All patients undergoing TUR-B showed transitional cell carcinoma in histological examination.

The control group showed median cFN plasma levels of 437 ng/ml (mean 652 ng/ml, range 181–2512 ng/ml, SD ± 374 ng/ml).

**Figure 1:**
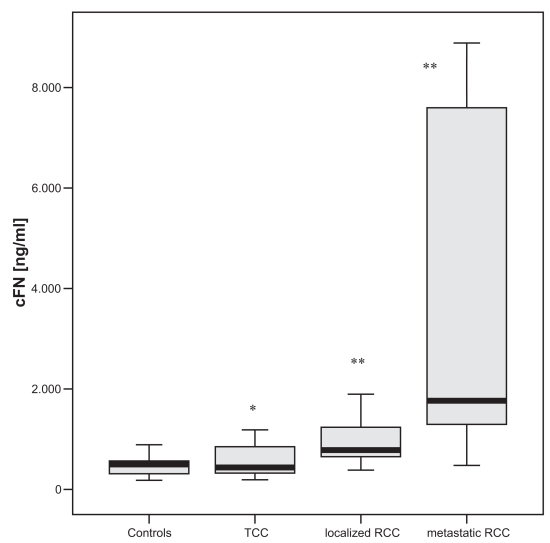
cFN plasma levels according to the different malignancy groups. * (p < 0.05) and ** (p < 0.005) vs. controls.

**Figure 2:**
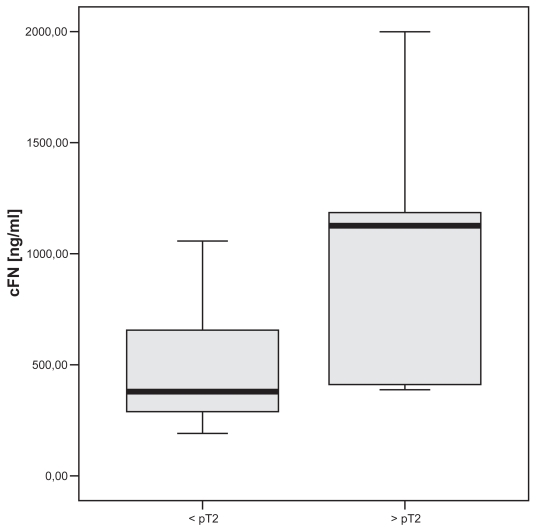
Significant higher levels of cFN in patients with muscle invasive TCC compared to non-muscle invasive TCC (p < 0.05).

The TCC group showed significant higher median cFN plasma levels with 521 ng/ml (p < 0.02, mean 836 ng/ml, range 274–3145 ng/ml, SD ± 447 ng/ml) compared to controls (Fig. 1). Subdividing the TCC group according to the UICC TNM-system of 2002 [24] in non-muscle invasive (< pT2, n = 20) and muscle invasive (> = pT2, n = 10) disease significant higher levels of plasma cFN (p < 0.05) could be detected in the muscle invasive group (median 1126 ng/ml, mean 1847 ng/ml, SD ± 664 ng/ml) compared to the non-muscle invasive group (median 379 ng/ml, mean 643 ng/ml, SD ± 267 ng/ml, Fig. 2).

**Figure 3:**
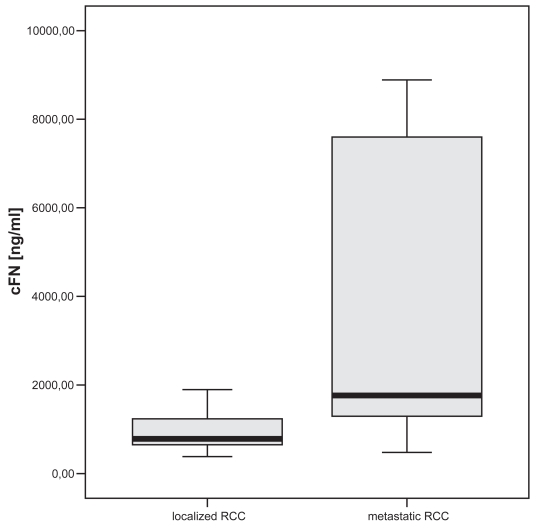
Significant elevated cFN levels in patients with metastatic RCC compared to localized stage of disease (p < 0.01).

Both the localized and metastatic RCC group showed significant higher levels of plasma cFN (p < 0.005) compared to controls (Fig. 1). According to tumor stage in patients with localized RCC no significant differences were noted. Patients with metastatic RCC (median 1764 ng/ml, mean 5727 ng/ml, 478–8967 ng/ml, SD ± 3403 ng/ml) showed significant higher levels of cFN (p < 0.01) compared to localized RCC (median 765 ng/ml, mean 1139 ng/ml, range 282–5271 ng/ml, SD ± 962 ng/ml, Fig. 3). The location and number of metastases showed no significant influence of cFN plasma levels.

In both malignancy groups there were no significant differences concerning nuclear grading (p > 0.5 respectively).

## Discussion

The goal of the present study was to evaluate the clinicial suitability of cFN for diagnosis and monitoring of patients suffering TCC and RCC. Until now for both urological malignancies no markers are present in clinical routine. Cellular FN, representing a multifunctional glycoprotein, seems to play an important role in local tumor progression and migration (Hynes and Yamada, 1982). In contrast to total FN, cFN is found in much smaller amounts in circulation and is not mainly synthesized by hepatocytes. Most available antibodies are directed against both forms. So discrimination between FN synthesized by hepatozytes and cFN is not possible and interpretation of the results is difficult when using conventional available immunoassays. The applied time-resolved fluorescence immunoassay has been thoroughly tested (Kropf and Gressner, 1995; Kropf et al. 1991). Using a monoclonal specific antibody it is possible to detect cFN without disturbing FN interference. The time-resolved fluorescence method is not easily available so in future the development of an assay based on photometrical detection is planned.

In TCC an insoluble matrix form of FN was found along the urothelian tissue, but studies revealed diverse results of FN expression in bladder cancer tissue (Kirkali et al. 2001; Sanchez-Carbayo, 2000; Cheng et al. 1988; Zhang et al. 2004; Fleischmann et al. 1993). FN seems to play a major role in TCC and the response to intravesical immunotherapy with Bacillus Calmette-Guérin (BCG) after tumor resection. But this role is completely unclear as reflected in contradictory findings concerning FN urine levels (Zhang et al. 2004; Fleischmann et al. 1993; Danisman et al. 2000). A possible explanation maybe the different detection methodologies used, their incomparability and patient dependent urine production with different consecutive FN concentrations. So detection in blood might be an additional tool and offer the chance to get more reliable and conclusive data.

We found significant elevated cFN levels in patients with TCC compared to controls pointing at the valuable but unclear role in TCC. The relationship between cFN and tumor progression is reflected in significant higher levels in patients with muscle invasive stage compared to non-muscle invasive stages. These findings are of interest for clinical routine. Eventually cFN plasma levels can play a role as supplementing tool beside histopathology and for monitoring during follow-up. A long term study is ongoing to clarify if initial elevated cFN plasma levels in non-muscle invasive TCC are accompanied with an earlier recurrence and/or progression of TCC. Additionally further investigations have to be performed to determine cFN plasma levels after successful primary therapy and during follow-up.

In RCC FN also seems to play an important role. Murata and co-workers showed an influence of FN on migration of different RCC cell lines in culture. SN12C-2 cell line with high metastatic potential migrated strongest when directed by a FN gradient (Murata et al. 1992). In addition Lohi and coworkers showed the ability of different RCC cell lines to secrete different FN isoforms (Lohi et al. 1996). The influence of ECM components, especially FN, on renal tumor cell invasion was clearly demonstrated by Brenner and co-workers (Brenner et al. 2000). Wunderlich and co-workers described a positive correlation of oncofetal FN expression with prognostic parameters and concluded that high oncofetal FN may indicate poor outcome (Wunderlich et al. 2002). Our investigation supports this impression but we could not find any correlation between cFN plasma levels and nuclear grading.

In RCC, a malignancy missing a suitable marker so far, we found significantly elevated plasma levels in patients suffering RCC compared to healthy controls. Additionally in metastatic stage of disease cFN levels are significantly increasing pointing out the possible role of cFN in advanced RCC. In the face of higher plasma levels at the time of curative treatment cFN can play a role as follow-up tool. Furthermore in metastatic disease cFN can be helpful as control tool for response to therapy. These topics have to be evaluated in further investigations.

Additionally one cannot exclude the existence and detection of abnormal circulating FN isoforms in the different malignancies leading to different results; so all results have to be discussed carefully (Castellani et al. 1986).

In summary due to the relatively high standard deviation of it’s plasma concentrations cFN seems not to be a suitable marker for screening and detecting TCC and RCC. However, in the face of significant elevated levels both in muscle invasive TCC and metastatic RCC cFN will be of high interest for monitoring patients during follow up after initial therapy: in TCC as a predictor of muscle invasive disease and in RCC as an indicator of recurrence and maybe to control response of therapy in metastatic stage of disease.

Our data on cFN as a marker of tumor progression for TCC and RCC encouraging further investigations with a larger number of patients before initial therapy and during follow-up to enlighten the future role of cFN for these urological malignancies.
